# Longitudinal relationship between social media and e-cigarette use among adolescents: the roles of internalizing problems and academic performance

**DOI:** 10.1186/s12889-023-17059-8

**Published:** 2023-10-31

**Authors:** Luxi Zhang, Song Harris Ao, Xinshu Zhao

**Affiliations:** 1 Department of Communication / Institute of Collaborative Innovation, University of Macau, Macau, China; 2 Department of Communication / Institute of Collaborative Innovation / Center for Research in Greater Bay Area, University of Macau, Macau, China

**Keywords:** E-cigarette, Social media, Adolescent health, Internalizing problems, Academic performance, Moderated mediation model

## Abstract

**Background:**

Prior research has investigated the influence of social media on e-cigarette use among adolescents, predominantly through the display of e-cigarette content and advertisements. However, the psychological mechanism underlying this relationship remains underexplored. This study aims to address the mediating effect of youths internalizing problems and elucidate the moderating effect of academic performance from a longitudinal perspective.

**Methods:**

Panel data from the Public Assessment of Tobacco and Health (PATH) Study Waves 3–5 (2015–2019) were utilized in this study. The sample consisted of 3,975 youths between the ages of 12 and 17 years old. A moderated mediation model was utilized for analyses.

**Results:**

Adolescents using social media more frequently in Wave 3 reported higher odds of internalizing problems in Wave 4 (*bp* = 0.061, *p* < .01), which led to more e-cigarette use in Wave 5 (*bp* = 0.029, *p* < .01). A negative moderating effect of academic performance was found in the association between internalizing problems and e-cigarette use (*bp*=-0.088, *p* < .05).

**Conclusions:**

Frequent social media use among young individuals leads to an increase in e-cigarette use through enhanced internalizing problems. However, adolescents who perform well academically exhibit higher resistance to e-cigarette use. Based on our findings, we recommend that tailored anti-e-cigarette campaigns and mental health interventions be used to target frequent social media users and academically struggling adolescents to prevent adverse health outcomes.

## Background

In recent decades, electronic cigarettes (e-cigarettes) have gained popularity among the young population in the United States. As per the Food and Drug Administration (FDA) and Centers for Disease Control and Prevention (CDC), in 2022, 1 in 10 middle and high school students reported currently using e-cigarettes [1]. In the U.S., conventional cigarette use among youth has declined, whereas the use of e-cigarettes has continued to increase [2]. The rise in e-cigarette use by youths has sparked widespread public health concerns. E-cigarette use may lead to short-term health harm, such as lung disease and mental issues [3, 4]. Additionally, e-cigarette use during adolescence can cause chronic and profound health damage, such as long-term nicotine dependence [4].

Due to the significant threat of e-cigarettes for youth health, scholars from various disciplines seek to identify the factors that influence youth e-cigarette use. Numerous theories posit risk factors linked to smoking behaviors [5–7]. These factors cover a wide range, with some focusing on proximal influences such as self-efficacy, social norms, attitudes, and internalizing issues [8–10]. Others focus on distal factors, including school influence and media influence [11–14]. Ultimate causes, such as personal traits and familial impacts, also feature prominently [15–17]. Therefore, tobacco use is the result of a complex, long-term process that is influenced by multiple predictors [18]. Some researchers address the need to incorporate multiple factors to comprehensively examine tobacco use. For example, Flay et al. (1994) introduced the Theory of Triadic Influence, a meta-theoretical framework that encompasses intrapersonal, interpersonal, and cultural-environmental influences [7, 19]. Understanding the collaborative impacts of multiple risk factors on e-cigarette use among youth is crucial for developing preventive measures.

Social media is becoming increasingly popular among youth and is considered a strong predictor of e-cigarette use [13, 14]. Most existing studies have focused on the effects of e-cigarette-related advertisements on social media [20–22]. Nevertheless, the underlying psychological mechanism governing the correlation between social media and e-cigarette use remains insufficiently understood. Recent studies have raised concerns over the potential of social media to induce internalizing problems in adolescents [23, 24]. These internalizing issues may, in turn, exacerbate the prevalence of e-cigarette use among adolescents [8, 25]. However, current observations lack sufficient evidence from longitudinal studies. Furthermore, while academic performance significantly impacts youth’s mental and behavioral health [26–28], its role in influencing adolescents’ internalizing problems and e-cigarette use is not fully understood. These research gaps highlight the necessity for further investigation.

### Social media, internalizing problems, and e-cigarette use: the psychological mechanism

Social media has become very prevalent among the youth population. These platforms enable individuals to freely communicate, develop and maintain relationships, and foster a sense of belonging [29, 30]. According to 2022 Pew Research data, 95% of adolescents between the ages of 13 and 17 have used social media, with the majority of them accessing these platforms on a daily basis. Furthermore, 54% of teenagers find it challenging to give up social media [31]. Social media may promote health by providing easy-access platforms for sharing and discussing health-related content, seeking health information, and gaining support [32–34]. However, excessive social media use may have adverse effects on adolescent health [23, 24, 35]. In a systematic review of 13 studies, it was found that increased time spent on social media is linked with higher rates of internalizing problems, such as depression and anxiety [24]. Furthermore, a small body of longitudinal studies has revealed a positive correlation between the frequency of social media use and internalizing problems [23, 36]. Several reasons account for the observed positive relationship, including addiction to social media, overwhelming unrealistic content, peer pressure and social isolation [23, 24]. Hence, we propose the first hypothesis:

H1. Social media use is positively associated with subsequent internalizing problems among youth. (*a* path)

Internalizing problems have been identified as a predictor of adolescent tobacco use. According to coping theory and affective regulation theory, individuals with mental health issues are inclined to use nicotine as a means of emotional numbing [37]. Empirical studies have consistently demonstrated that higher levels of internalizing problems lead to increased use of e-cigarettes [8, 25]. For instance, a longitudinal investigation revealed that youth with elevated internalizing problems were more likely to engage in greater subsequent e-cigarette use [8]. Therefore, we propose the following hypothesis:

H2. Internalizing problems are positively associated with subsequent e-cigarette use among youth. (*b* path)

The mediating role of internalizing problems has been addressed in several adolescent health studies [18, 38, 39]. Internalizing problems are recognized as an essential psychological mediating factor in the process from environmental influence to problematic behaviors [18, 38]. For example, it has been demonstrated that internalizing problems mediate the association between childhood maltreatment and smoking behaviors among adolescents [38]. Moreover, a cross-sectional investigation revealed a positive mediating effect of internalizing problems on the link between social media use and smoking status [39]. Building upon these findings, we pose the following research question to examine the mediating effect of internalizing problems on the association between social media use and e-cigarette use:

RQ1. What is the mediating effect of internalizing problems on the association between social media use and e-cigarette use? Positive or negative? (a*b path)

Extensive research has examined the direct association between social media use and the consumption of e-cigarettes by teenagers. Various empirical studies have established a link between the advertisements of e-cigarettes on social media and enhanced e-cigarette use among youth [13, 14, 20–22]. Due to their perception that e-cigarettes are harmless and trendy, adolescents are vulnerable to deceptive marketing messages [20–22]. Thus, we propose the following hypothesis:

H3. Social media use is positively associated with subsequent e-cigarette use among youth. (*d* path)

### Academic performance: a risk or protective factor?

Schooling plays a crucial role in the daily lives of adolescents and significantly influences their behaviors [40]. According to the Social Development Model (SDM), adolescents acquire behavioral patterns, whether prosocial or antisocial, from socializing agents such as schools [26–28]. Schools shape adolescent behaviors by establishing normative environments and evaluating outcomes, such as academic performance [41]. Academic performance serves as a comprehensive indicator of personal attributes such as confidence and self-control, peer network dynamics, and the degree of adherence to social norms [42]. Due to its complexity and characteristics, academic performance becomes a significant factor influencing the health of adolescents as early as Grade 6 or 7 [43, 44]. According to the SDM framework, academic performance is both protective and risky, with varying effects on adolescents’ mental and behavioral health [26–28]. It hypothesizes that academic performance has a moderating effect on risk exposure [26]. Specifically, high-achieving students may exhibit greater resilience to risks than academically challenged students because the former have higher levels of self-control and stronger peer support [42, 45].

Empirical studies consistently demonstrate that academic performance is one of the foremost influences associated with both mental health and substance use among adolescents [43, 46, 47]. However, the impact of good and poor academic performance differs concerning mental well-being and tobacco use in youth. Research has revealed that academic performance is a negative predictor of internalizing problems among youth [46]. Those who excel academically exhibit resilience toward internalizing problems [48], whereas adolescents with poor academic performance are more vulnerable to internalizing problems. Such academic incompetence may enhance adolescents’ self-deprecation and impede their social standing [49]. Similarly, there is a negative association between academic performance and youth smoking behaviors, including the use of e-cigarettes. For instance, a cohort study found that poor academic performance in high school can predict subsequent smoking behaviors [50]. A cross-sectional study demonstrated that low grades are linked to e-cigarette use [47]. Conversely, students who perform well tend to manifest higher resistance to tobacco use [42, 45].

The wide variation in academic performance is well documented [26–28, 40, 41], suggesting its significant potential as a moderator. However, empirical investigations have rarely explored this moderating effect. To comprehensively understand the moderating mechanism of academic performance, we propose that it may moderate the association between internalizing problems and e-cigarette use. Hence, we pose our second research question as follows:

RQ2: What is the moderating effect of academic performance on the association between internalizing problems and e-cigarette use? Positive or negative?

**Fig. 1:**
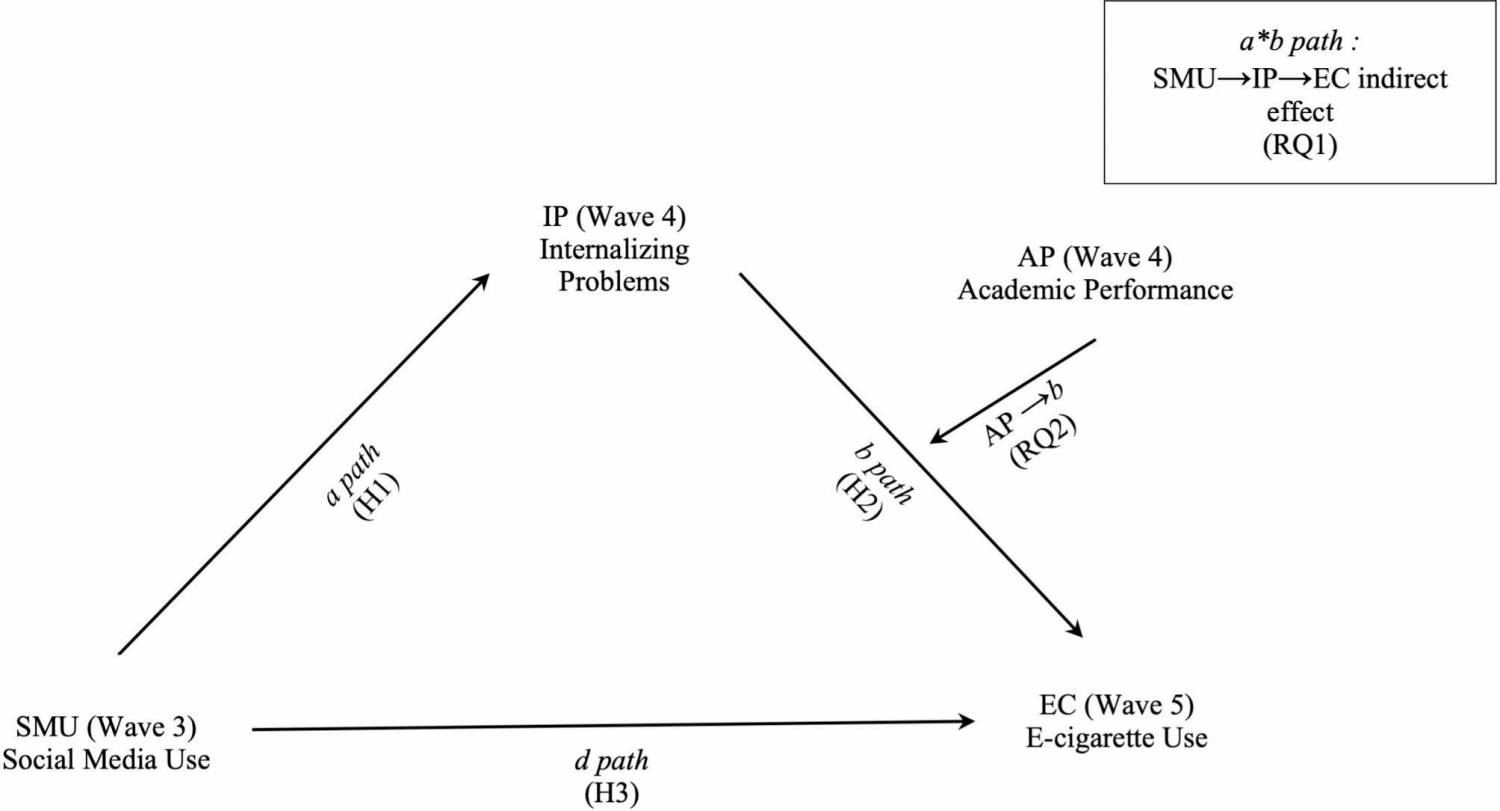
Conceptualization of the model

In summary, this study builds upon prior research and seeks to address three research gaps. First, it employs a moderated mediation model to examine the longitudinal relationships among social media use, internalizing problems, academic performance, and e-cigarette use among youth. Second, it explores the mediating mechanism that links social media use to e-cigarette consumption. Last, it investigates academic performance as a potential moderator and its influence on youth e-cigarette use. Figure 1 presents the conceptual model that outlines all the hypotheses.

## Methods

### Data source and sample

The present study utilized data from Waves 3 to 5 of the Population Assessment of Tobacco and Health (PATH) study, which is a longitudinally designed, nationally representative cohort study carried out jointly by the National Institutes of Health (NIH) and the Food and Drug Administration (FDA). The purpose of the PATH study is to monitor tobacco use and its associated health consequences among both youths and adults across the United States [51]. The follow-up rates for the Wave 1 youth cohort were 83.3%, 79.5%, and 72.3% for Waves 3 (2015–2016), Wave 4 (2016–2018), and Wave 5 (2018–2019), respectively. For the present study, data across these three waves were pooled, and youth from 12 to 17 years old with complete data on key variables (social media use in Wave 3, internalizing problems in Wave 4 and e-cigarette use in Wave 5) were selected, resulting in a total sample of 3,975 respondents. Listwise deletion was applied for nonvalid responses in the regression analyses.

### Measures

The dependent variable *e-cigarette use* in Wave 5 is the frequency of usage, which is measured by the number of days the respondents reported using e-cigarettes in the past 30 days (0–30) [13]. Although e-cigarette quantity is also an important measure [52, 53], its validity and reliability remain questionable due to the complexity and variations among different e-cigarette devices [54, 55]. Therefore, following previous literature [13, 56], the frequency of e-cigarette usage in the past 30 days was used as the measurement in this study.

The independent variable *social media use* in Wave 3 measures the frequency of social media use. The response was a 7-point Likert scale that included (1) Never; (2) Less often; (3) Every few weeks; (4) 1–2 days a week; (5) 3–5 days a week; (6) About once a day; and (7) More than once a day [13]. This is a continuous variable (1–7), and a larger number indicates more frequent social media use.

The mediator *internalizing problems* in Wave 4 was a compound variable applying Global Appraisal of Individual Needs-Short Screener (GAIN-SS) [57]. Widely regarded for its reliability and validity [8], the GAIN-SS effectively measures symptoms associated with internalizing problems. The respondents were asked whether they had experienced the following symptoms of internalizing problems in the last year (Cronbach’s α = 0.871): (1) feeling very trapped, lonely, sad, blue, depressed, or hopeless about the future; (2) sleep trouble, such as bad dreams, sleeping restlessly, or falling asleep during the day; (3) feeling very anxious, nervous, tense, scared, panicked, or like something bad was going to happen; and (4) becoming very distressed and upset when something reminded you of the past. Each item was 0 (*no*) or 1 (*yes*), and the compound variable ranged from 0 to 4 [58].

The moderator *academic performance* in Wave 4 consisted of the respondents’ parents reporting their academic grades of the previous year. Academic performance was categorized into nine levels: (1) mostly F’s; (2) D’s and F’s; (3) mostly D’s; (4) C’s and D’s; (5) mostly C’s; (6) B’s and C’s; (7) mostly B’s; (8) A’s and B’s; and (9) mostly A’s. Academic performance is a continuous variable (1–9), where a larger number indicates better academic performance [2].

In the regression analyses, the sociodemographic variables age, gender, race, and household income from Wave 3 were incorporated as covariates. Furthermore, self-reported mental health, physical health, and family smoke exposure were controlled for potential factors related to e-cigarette use. In addition, to mitigate the impact of autoregressive effects, internalizing problems and e-cigarette use in Wave 3, e-cigarette use in Wave 4 were controlled. Table 1 presents descriptive statistics for all variables.

**Table 1:** Descriptive statistics of the independent, dependent, mediating and controlling variables (n = 3,975)

*Dependent variable*	
E-cigarette use in past 30 days in Wave 5 (0 ~ 30, Mean ± SD)	1.75 ± 5.97
*Independent variable*	
Social Media Usage Wave 3 (1 ~ 7, Mean ± SD)	5.91 ± 1.62
*Mediating variable*	
Internalizing Problems in Wave 4 (0 ~ 4, Mean ± SD)	1.91 ± 1.62
*Moderating variable*	
Academic Performance in Wave 4 (1 ~ 9, Mean ± SD)	7.36 ± 1.67
*Covariates*	
Age in Wave 3 (n. %)	
12–14	3,882 (97.7)
15–17	93 (2.3)
Gender in Wave 3 (n. %)	
Female	2.048 (51.5)
Male	1,914 (48.2)
Race in Wave 3 ((n. %)	
White alone	2,470 (62.1)
African American alone	644 (16.2)
Other	625 (15.7)
Household income in Wave 3 (n. %)	
Less than $10,000	312 (7.8)
$10,000 to $24,999	670 (16.9)
$25,000 to $49,999	913 (23.0)
$50,000 to $99,999	907 (22.8)
$100,000 or more	933 (23.5)
Self-perceived physical health in Wave 3 (1 ~ 5, Mean ± SD)	4.14 ± 0.93
Self-perceived mental health in Wave 3 (1 ~ 5, Mean ± SD)	3.72 ± 1.18
Exposed to Family secondhand smoke in Wave 3 (n. %)	
Yes	1,307 (32.9)
No	2,593 (65.2)
Internalizing Problems in Wave 3 (0 ~ 4, Mean ± SD)	1.76 ± 1.56
E-cigarette use in past 30 days in Wave 3 (0 ~ 30, Mean ± SD)	0.09 ± 1.11
E-cigarette use in past 30 days in Wave 4 (0 ~ 30, Mean ± SD)	0.15 ± 1.48

Notes: a SD stands for standard deviation.

### Data analysis

SPSS software (v26) was used to analyze data. Descriptive analyses were initially conducted to summarize the key variables, followed by the Pearson correlation test to explore relationships among continuous variables. ANOVA was performed to examine demographic differences, such as gender. The study also employed the SPSS macro PROCESS [59] Model 4 to identify the mediating effect of internalizing problems. Furthermore, PROCESS Model 14 was utilized to identify the moderating effect of academic performance on the association between internalizing problems and e-cigarette use. To compare the effect size of predictive factors, this study further adopted the *percentage coefficient (b*_*p*_*). B*_*p*_ is a *b* coefficient when the dependent variable and independent variable are each linearly transformed to a percentage scale (0–1) [60].

## Results

### Preliminary analyses

Table 1 presents the descriptive statistics. Of the respondents, 51.5% were female. Regarding race, 62.1% identified as white. A majority (69%) of the participants reported an annual household income above $25,000. In Wave 3, more than half (55%) of the respondents reported using social media at least once a day. Additionally, approximately one-third (33%) of the respondents reported exposure to secondhand smoke in their families. In Wave 4, a substantial proportion (67.2%) of the adolescents reported experiencing at least one internalizing problem symptom in the past year. Owing to the longitudinal characteristics of panel data, the age composition of cohorts changed over time. In Wave 3, 97.7% fell within the 12–14 age group, while 2.3% belonged to the 15–17 age group. Subsequently, during the following two waves, the adolescent cohorts matured. The 15–17 age group grew to 36.6% in Wave 4 and further increased to 97.4% in Wave 5.

The Pearson correlation existed among most of the key variables. Notably, a positive correlation was observed between social media use and internalizing problems, as well as e-cigarette use. Conversely, a negative correlation was observed between academic performance and e-cigarette use. ANOVA demonstrated that there were differences in social media use and internalizing problems between males and females.

### Testing mediation

**Fig. 2:**
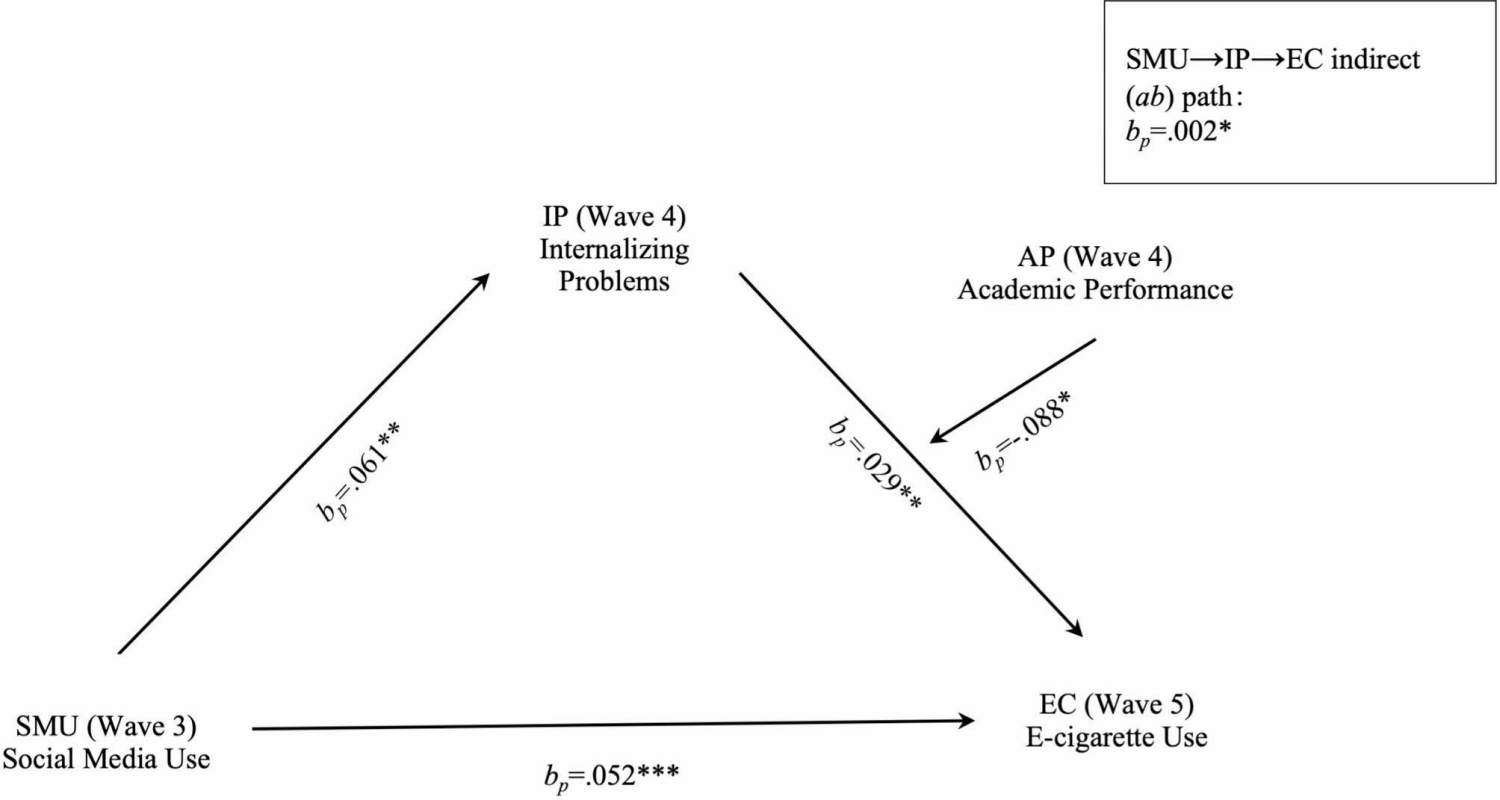
Effect of SMU on EC mediated by IP and moderated by AP *Notes:* Path indicators are regression coefficients in parentheses, (*b*_*p*_). *p < .05. **p < .01. ***p < .001

H1 postulated a positive link from social media use in Wave 3 to internalizing problems in Wave 4. As shown in Table 2 and Fig. 2, the association was positive and passed the statistical threshold (*b*_*p*_=0.061, *β* = 0.041, *p* < .01), therefore supporting H1.

**Table 2:** Summary of mediation and moderation effects

Mediation pathway	*bp*	*β*	SE	95% CI
*a* path: SMU (Wave 3)→IP (Wave 4)	0.061**	0.041**	0.022	[0.019, 0.104]
*b* path: IP (Wave 4)→EC (Wave 5)	0.029**	0.058**	0.010	[0.009, 0.048]
a*b path: SMU (Wave 3)→IP (Wave 4)→EC (Wave 5)	0.002*	0.002*	0.001	[0.000, 0.004]
*d* path: SMU (Wave 3)→EC (Wave 5)	0.052***	0.070***	0.012	[0.027, 0.076]
**Moderation pathway**				
IPxAP (Wave 4)→*b* path	− 0.088*	− 0.153*	0.038	[-0.162, − 0.015]

*p < .05; **p < .01; ***p < .001

*bp*: percentage coefficients; *β*: Standardized beta; CI stands for confidence interval. SMU: social media use; IP: internalizing problems; EC: e-cigarette use in last 30 days; AP: academic performance

All model controlling for age, gender, race, household income, self-reported physical health, self-reported mental health, family secondhand smoke exposure in Wave 3; internalizing problems in Wave 3; current e-cigarette use in Wave 3 and Wave 4

H2 predicted a positive association between internalizing problems in Wave 4 and e-cigarette use in Wave 5. There was indeed a statistically acknowledged positive effect (*b*_*p*_=0.029, *β* = 0.058, *p* < .01). H2 was thus supported.

RQ1 asked the mediating effect of internalizing problems in Wave 4 on the association between social media use in Wave 3 and e-cigarette use in Wave 5. The results showed a statistically positive mediation effect (*b*_*p*_=0.002, *β* = 0.002, bootstrap 95% CI ranges [0.000, 0.004], Sobel test *p* < .05).

H3 predicted a positive direct effect from social media use in Wave 3 to e-cigarette use in Wave 5. As shown in Table 2, the direct path was statistically positive (*b*_*p*_=0.052, *β* = 0.070, *p* < .001). H3 was supported.

### Testing moderation

The second research question asked about the moderating effect of academic performance on the association between internalizing problems and e-cigarette use. As shown in Table 2, the moderating effect was negative and statistically acknowledged (*bp*=-0.088, *β*=-0.153, *p* < .05).

**Fig. 3:**
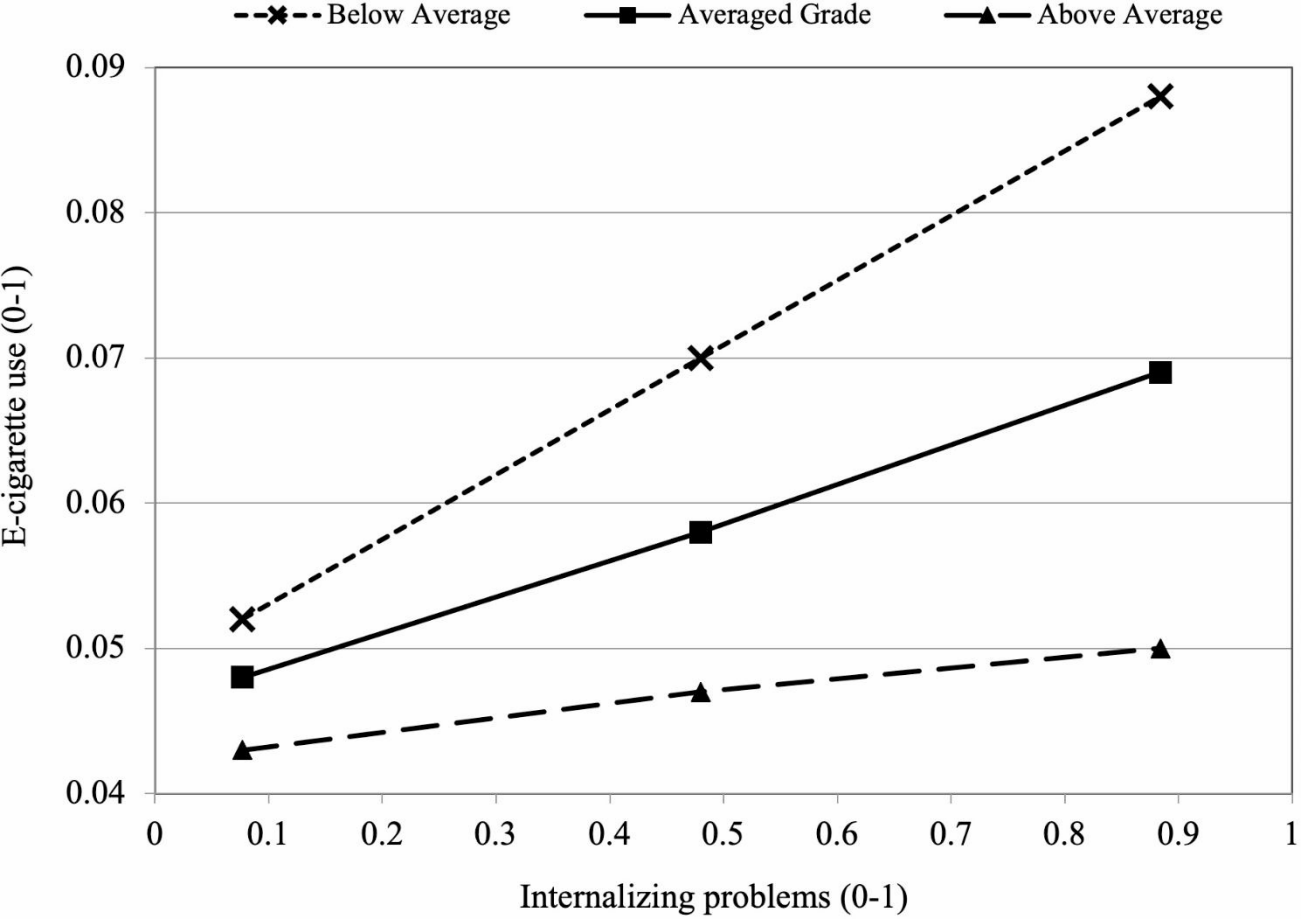
Moderation effect of academic performance on IP→EC path (*b* path)

Figure 3 illustrates the moderating role of academic performance wherein the positive correlations between internalizing problems and e-cigarette use are apparent across all three groups (below average, average, and above average). Nevertheless, this trend attenuates considerably among the higher academic performance group. Adolescents who perform well academically exhibit greater resilience compared to those with poor school performance.

## Discussion

This research presents a moderated mediation model that consolidates multiple factors linked with youth e-cigarette consumption. The study provides preliminary evidence for the psychological mechanism underlying the relationship between social media and e-cigarette use. Furthermore, the investigation reveals the moderating effect of academic performance, which expands the literature.

### Mental health matters: addressing the mediating role of internalizing problems

Consistent with previous findings [8, 13, 14, 20–22, 25], social media use and internalizing problems were found to be risk factors in predicting e-cigarette use among adolescents. Furthermore, this study uncovered a less explored phenomenon, revealing that internalizing problems mediated the association between social media use and e-cigarette use. Despite increased focus on the negative mental health outcomes associated with social media use in recent years [23, 24, 36], the subsequent risky behaviors due to such internalizing problems have received less attention.

In this study, the nature of longitudinal data was leveraged, and it was found that adolescents predominantly aged 12 to 14 years old who frequently used social media reported higher odds of developing internalizing problems in the subsequent 1–3 years, which in turn was associated with increased e-cigarette use in another next 1–3 years. Consistent with previous research [18, 38], our findings reinforce the concept that internalizing problems act as a mediating mechanism between environmental influences and problematic behaviors. These findings elucidate the complex and long-term process of youth e-cigarette use, which includes both distal and proximal factors. In this enduring process, social media use and internalizing problems are interrelated and combined as more potent negative contributors to youth e-cigarette use [18].

### The moderating effect of academic performance

This study demonstrated the moderating role of academic performance. The findings built on the SDM and corroborated previous empirical studies [26–28, 42, 45, 47, 50]. Moreover, this study revealed that adolescents with different levels of academic performance (below average, average, and above average) exhibit different reactions to using e-cigarettes triggered by internalizing problems. Compared to those who experience academic success, adolescents who struggle academically tend to have more internalizing problems and are more likely to use e-cigarettes. In other words, the positive association between internalizing problems and e-cigarette use is more pronounced among adolescents with poorer academic performance. It is plausible that youth with better school performance possess certain traits, including self-confidence, competence in value judgments, and stronger attachments to society [42, 45]. All these features increase their resistance to risky behaviors.

In contrast, the situation differs for adolescents with poor grades. Consistent with prior research [47, 49, 50], it was found in the present study that academic failure may exacerbate e-cigarette use among adolescents with internalizing symptoms, thereby functioning as a risk factor. Students with poor academic performance may not gain satisfaction or receive positive feedback. Instead, these students with poor grades may progressively lose their attachment to school and fall into a vicious cycle [26–28]. Additionally, poor academic performance may place students in a social environment with low-achieving peers, elevating the likelihood of subsequent delinquent behaviors [49, 50]. Understanding the moderating role of academic performance is vital for policymakers and educators to develop tailored strategies for different groups.

### Theoretical and clinical implications

This study contributes several theoretical advancements. First, while previous literature has primarily examined the direct association between social media and e-cigarette use [13, 14, 20–22], this study extends the literature by revealing the psychological mechanism underlying this relationship. Mental health professionals should place greater emphasis on the internalizing problems of adolescents, especially those who use social media frequently. Second, this study provides longitudinal evidence that academic performance has moderating effects. Adolescents’ academic performance is not only directly linked to their attitudes and behaviors regarding e-cigarettes but also moderates the relationship between risk factors and e-cigarette use. The findings of this study validate the concepts of the SDM, hypothesizing that factors containing protective features may moderate the effects of risk exposure [26, 27]. Third, this study extends the literature by incorporating multiple risk factors associated with e-cigarette use and demonstrating the long-term and complex relationships among these factors. Our findings encourage scholars to investigate multiple factors accounting for youth delinquent behaviors from longitudinal perspectives in future studies.

This study also has important practical implications for public health clinicians, educators, media, and manufacturers. First, frequent social media use was found to result in subsequent internalizing problems and e-cigarette use among youth. Therefore, the guidance of appropriate social media use and anti-addiction education should be implemented to curb this issue [23]. Additionally, social media platforms may need to enforce stricter restrictions on advertising, promoting, and selling e-cigarettes to adolescents while also monitoring the commercial activities of e-cigarette manufacturers [61]. Second, adolescence is a vulnerable period in which young people are more prone to mental health issues and risky behaviors. The incidence of mental health issues among U.S. youth has increased [23], and our study revealed that 67.2% of adolescents reported experiencing at least one internalizing problem symptom. Thus, it is essential for families, schools, communities, and mental health professionals to provide adequate support for young people’s mental health needs. Third, classroom-based tobacco education is encouraged to help students assess the risks and regulations surrounding e-cigarettes. Encouraging adolescents to participate in community-based advocacy activities, such as developing e-cigarette regulation materials for storeowners in the community, is also recommended [62]. Fourth, given that adolescents with higher academic performance show greater resilience to risk behaviors, measures that encourage students with poor grades to increase their investment and involvement in education should be implemented. Such measures may not only improve their academic performance but also reduce e-cigarette use. Finally, e-cigarette manufacturers should assume social responsibilities to protect adolescents and avoid inappropriate marketing strategies. For instance, they should refrain from promoting e-cigarettes as coping mechanisms for mental health issues and developing targeting plans aimed at youth [63].

### Limitations

This study has several limitations. First, the reliance on self-report survey data may lead to recall biases. Second, while the findings of this study suggest negative associations with social media use, it is important to acknowledge the potential positive effects of social media. Specifically, the study measures the frequency of social media use; however, it is possible that the specific use of social media may lead to different health outcomes [23]. Future research should therefore investigate the purpose of social media use and its subsequent impact on health. Additionally, although the frequency of social media use serves as one indicator of social media addiction [64], there is a gap between frequency of use and the presence of social media addiction [65, 66]. Future studies should therefore explore the health consequences of social media addiction using validated measurements [66]. Third, the variable e-cigarette use in this study measures the frequency of e-cigarette use. As previously mentioned, the quantity of e-cigarette use is equally significant [52]. It is strongly recommended that future studies further examine e-cigarette use comprehensively by incorporating both frequency and quantity with reliable measurement tools.

## Conclusion

In summary, this study examined the relationships among social media use, internalizing problems, academic performance, and e-cigarette use among youth in large longitudinal samples. The results disclosed a psychological pathway linking social media use and e-cigarette consumption, underscoring the mediating role of internalizing problems. Furthermore, using the SDM as a basis, this research provided evidence that academic performance could moderate the association between internalizing problems and e-cigarette use. Those who exhibited satisfactory performance demonstrated greater resistance to e-cigarette consumption. Based on an understanding of the complex interplay among these factors in relation to youth e-cigarette use, we suggest that educational strategies, interventions aimed at mental well-being, and anti-tobacco campaigns aimed at adolescents with frequent social media use and poor academic performance could have substantial potential to stem e-cigarette consumption among teenagers.

## Data Availability

Public use data available via the National Addiction and HIV Data Archive Program https://www.icpsr.umich.edu/web/NAHDAP/studies/36498.

## Abbreviations

E-cigarette: electronic cigarette
PATH: the Public Assessment of Tobacco and Health study
SD: standard deviation
SDM: social development model
*b*_*p*_: percentage coefficient
